# Exercise for Prevention and Relief of Cardiovascular Disease: Prognoses, Mechanisms, and Approaches

**DOI:** 10.1155/2019/3756750

**Published:** 2019-04-09

**Authors:** Danyang Tian, Jinqi Meng

**Affiliations:** ^1^Department of Physiology, Hebei Medical University, Shijiazhuang, China; ^2^Department of Sports, Hebei Medical University, Shijiazhuang, China

## Abstract

This review is aimed at summarizing the new findings about the multiple benefits of exercise on cardiovascular disease (CVD). We pay attention to the prevalence and risk factors of CVD and mechanisms and recommendations of physical activity. Physical activity can improve insulin sensitivity, alleviate plasma dyslipidemia, normalize elevated blood pressure, decrease blood viscosity, promote endothelial nitric oxide production, and improve leptin sensitivity to protect the heart and vessels. Besides, the protective role of exercise on the body involves not only animal models in the laboratory but also clinical studies which is demonstrated by WHO recommendations. The general exercise intensity for humans recommended by the American Heart Association to prevent CVD is moderate exercise of 30 minutes, 5 times a week. However, even the easiest activity is better than nothing. What is more, owing to the different physical fitness of individuals, a standard exercise training cannot provide the exact treatment for everyone. So personalization of exercise will be an irresistible trend and bring more beneficial effects with less inefficient physical activities. This paper reviews the benefits of exercise contributing to the body especially in CVD through the recent mechanism studies.

## 1. Cardiovascular Diseases: Prevalence and Risk Factors

CVD is a class of diseases which are related to the heart or blood vessels including stroke, heart failure, hypertension, coronary artery diseases, heart arrhythmia, peripheral artery disease, and atherosclerosis [1]. Individuals with CVD are found to have the accompanying raised blood pressure, elevated glucose, smoking, obesity, lack of exercise, excessive alcohol consumption, and dyslipidemia. Fortunately, CVD can be properly managed and prevented by controlling blood pressure, glucose, lipid, smoking, and alcohol drinking and through lifestyle modifications for sleep, emotion, exercise, and diet, which are called SEED intervention [2]. With the aging of population in the world, CVD has become the leading cause of death globally. Approximately 17.9 million deaths in 2015 were caused by CVD in the world [3]. The percentage of Chinese older than 60 has increased to 16% in 2015 which directly leads to a consequence that cardiovascular disease is becoming the leading cause of death in China [4]. Multiple risk factors contributing to CVD include obesity, high blood pressure, diabetes, aging, male sex, metabolic syndrome, and physical inactivity.

Determined by body mass index (BMI), obesity refers to the condition of those who have over 30 BMI and overweight includes persons with BMI more than 25. The prevalence among people of obesity has exceeded 50% in most countries and is increasing in both adults and children over the past few decades worldwide [5]. Obesity is found to increase blood volume, CRP, and TNF*α* which cause cardiac remodeling and inflammation. Obesity also aggravates the risks of high blood pressure, stroke, myocardial infarction (MI), and insulin resistance, which are all risk factors of CVD [6]. Furthermore, mortality and morbidity of CVD have been shown increasing in overweight populations, especially in those with abdominal obesity [7].

As the most common form of diabetes, type 2 diabetes is a chronic metabolic disease which is characterized by high blood glucose, low insulin sensitivity. With the incidence of diabetes continuing to rise, the number of patients diagnosed with diabetes has swelled from 30 million in 1985 to 382 million in 2014, and scientists predict 592 million people will have diabetes by 2035, almost 1 in 10 persons suffering from this disease [8]. Besides affecting aging people, this disease also influences an increasing number of young populations and even children [9]. Lots of evidences and studies demonstrate that type 2 diabetes acts as an independent risk factor for CVD. The patients who are diagnosed with type 2 diabetes have a worse prognosis and therapeutic effects of CVD compared with those without diabetes. CVD death rates with diabetes in the United States adults are 1.7 times higher than those without diabetes [10]. The huge medical care burden of type 2 diabetes generally attributes to vascular complications like MI, hypertension, peripheral vascular disease, and coronary artery disease [11].

Aging is a gradual, systematic, irreversible, degenerative process in the body, which results in weakness, disease, and even death. As there is an average increasing lifespan for humans, there will be approximately 20% of the population over 65 by 2030. Aging is an inevitable and important determinant for CVD which leads to the decline of mitochondrial functions, excess reactive oxygen species (ROS) production, and disorder of Ca^2+^ levels [12]. Aging is relevant to the progressive damage in various physiological processes and increases incidence of atherosclerosis, hypertension, and stroke [13], thus inducing an elevating risk of cardiac and arterial systematic disorders. Studies show parts of the key genes regulating lifespan including AMPK, m-TOR, and IGF-1; sirtuins are closely related to CVD progress [14].

Epidemiologists demonstrate the different CVD occurrence rates between males and females. Clinical studies show that the onset of heart attack is delayed 9 years in women compared with men [15]. In a cross-sectional survey on hospitalized patients with coronary artery disease, women were found 3.1 years older than men [16]. It is widely believed that estrogen is the prominent protective element for females [17]. Besides, women tend to have a better awareness of weight measurement and their waist circumference if they have central obesity, which would lower the risk of adiposity and dyslipidemia. Smoking and alcohol drinking are severe risk factors for CVD, which are more common lifestyle behaviors in men compared to women [18].

Metabolic syndrome is a condition characterized by low high-density lipoprotein (HDL), high triglycerides (TG), high blood pressure, high blood glucose, and central obesity, associating with the risks of developing to CVD and type 2 diabetes. Studies show that metabolic syndrome affects 30-40% of people older than 65 by doubling the risk of getting CVD [19]. Low HDL and high TG are associated with elevated levels of low-density lipoprotein (LDL), which increase the risk of having atherosclerosis [20]. High glucose links to dysfunctions of glucose uptake and catabolism and induces high oxidative stress, dysfunction of endothelial cells. High blood pressure leads to cardiac and vascular injury. Central obesity is related to maldistribution of free fatty acid, overproduction of inflammatory molecules, and leptin resistance [21].

Physical inactivity has been identified as the fourth risk factor of death worldwide, leading to approximately 3.2 million deaths annually. Various studies show an obvious dose-response relationship between increased physical activity and decreased occurrence rate of CVD including reduced blood pressure, body weight, ox-LDL, and elevated glucose tolerance. A systematic review estimates that the lack of exercise leads to 6% of coronary heart disease occurrence worldwide. Deficiency in physical activity leads to obesity, increasing endogenous inflammatory molecules and coagulation factors. In addition, there is coordinated protective effects to decrease the overall risk of incident CVD by exercise [22, 23]. So having a healthy diet, avoiding smoking, and keeping regular physical activity are the three pieces of advice the WHO recommend to avoid CVD.

## 2. Mechanisms of Action for Physical Exercise

Many considerable evidences support the therapeutic and protective effects of exercise on the body, including improvement of insulin sensitivity of diabetic mice, attenuating sympathetic activity, arterial pressure, and heart rate in the spontaneously hypertensive rats [24]. Mitochondrial biogenic response, components of the electron transport chain, mtDNA, and related lipid metabolic pathways are all increased after exercise training [25]. Here, we talk about the benefits of exercise on cardiovascular disease from the following aspects.

### 2.1. Insulin Sensitivity and Blood Glucose Control

Type 2 diabetes mellitus is a kind of chronic disease characterized by obesity, hyperglycemia, impaired insulin secretion, and insulin resistance [26]. Studies show that diabetic rats with exercise training present reduced body weight, decreased TG levels, and diminished blood glucose levels compared with those sedentary rats [27, 28]. PPAR*γ* known as “energy-balanced receptor,” is well studied in metabolic disorders. Carnitine palmitoyl transferase-1 (CPT-1) mainly transports fatty acids into mitochondria for medium-chain acyl-CoA dehydrogenase (MCAD) catalyzing *β*-oxidation. Through upregulating PPAR*γ* and its target genes, CPT-1 and MCAD, exercise alleviates hepatic steatosis, promotes glucose uptake, and improves insulin sensitivity in nonalcoholic fatty liver disease mice [29]. Exercise stimulates the translocation of glucose transporter type 4 (GLUT4) from the cytoplasm to the cell membrane, thus promoting glucose uptake and improving insulin resistance [30]. Besides, improved insulin sensitivity is independent with exercise modality. High or low intensity of exercise and aerobic or anaerobic training lead to improvement in glucose clearance curve and insulin sensitivity [31].

Besides improving insulin sensitivity, exercise facilitates glucose uptake and usage via insulin-independent mechanisms. Once glucose enters muscular and adipose cells, it will be phosphorylated by hexokinase which is an irreversible catalytic reaction to form glucose 6-phosphate (G-6-P) that cannot diffuse back out of cells. Glycolysis and glycogenesis started from G-6-P and promote glucose uptake by cells and usage in cells to affect blood glucose. Exercise increases G-6-P level in the skeletal muscles accompanying increased GLUT4, hexokinase level, and glycogen synthase activity, which finally improve glucose tolerance and decrease blood glucose level [32].

### 2.2. Lipid Profile

Cholesterol is a soft waxy fat that our body needs to function well. But too much cholesterol will become risk factors for human diseases like heart disease, stroke, and atherosclerosis [33]. For those who have been diagnosed with diabetes, heart disease, and stroke or people who are taking medicine to control cholesterol level, taking cholesterol test every year is necessary [34]. Generally, a cholesterol test includes total cholesterol, LDL, HDL, and TG [35]. Standard management strategies like drug therapy and diet control are generally used to lower serum cholesterol to prevent heart disease. However, some people are insensitive to statins or cannot tolerate statins. Hence, other ways need to replace or be used together with statins. More and more evidences support aerobic exercise as a positive method for alleviating plasma dyslipidemia and improving the prognosis of cardiovascular diseases [36]. Through using meta-analyses to investigate exercise and lipid profiles, Pedersen et al. concluded that exercise led to benefits of physical health [37]. A prospective cohort study lasting for 10 years about exercise and lipid metabolism shows that the risk of mortality is significantly reduced by combining statins with exercise, especially compared to other therapy alone [38]. Comparing with LDL and TG, HDL is more sensitive to exercise. Studies indicate that HDL is increasing more or less both in humans and rats after exercise [39, 40]. For the “bad” cholesterol LDL, the effects of exercise reduce the serum levels significantly in rats [40]. However, the effects are not consistent in humans, which may be due to the different dietary habits and living conditions [41, 42]. It is strongly accepted and reported that exercise leads a high requirement of energy which induces decreasing of plasma TG concentrations [43].

### 2.3. Blood Pressure

Blood pressure is elicited by the force exerted by the blood against the blood vessels, which depends on the ejection of the heart and resistance of the blood vessels. Hypertension is another name of high blood pressure, a disease related to heart attack, stroke, heart failure, and other problems [44]. Exercise always leads to a postexercise hypotension, and both normotensive and hypertensive persons experience a transient reduction in blood pressure. The reduced magnitude may achieve the point wherein patients with hypertension recover to the normal blood pressure levels. In a meta-analysis, they investigated the effects of acute exercise on blood pressure response. There were significant changes, reduction of 4.8 mmHg for systolic blood pressure (SBP) and 3.2 mmHg for diastolic blood pressure (DBP). The epidemiological study demonstrates that 2 mmHg decline in SBP leads to 6% reduction of stroke mortality and 4% reduction of coronary heart disease mortality, and a decrease of 5 mmHg causes the reduction of mortality of these diseases by 14% and 9%, respectively [45]. So the meta-analysis results confirm the undoubted place of noninvasive therapy method, acute exercise.

The transient reduction only lasts for a few hours and would recover after rest. However, the benefits of physical activity cannot be ignored because of chronic treatment of exercise showing significant changes among subjects. The vasodilator activity leads to the decrease of blood pressure. On the contrary, increased blood pressure is caused by vasoconstriction. Moderate-intensity exercise causes a vasodilatory response and decreases the vasoconstricting response and lipid in rat aortas, which exhibits a decrease in diastolic blood pressure [46, 47]. In addition to the vascular tone, exercise decreases blood pressure through lowering oxidative stress and inflammation levels. In the spontaneous hypertension rats (SHR), exercise normalizes the increased collagen deposition and diminished fenestra size in the internal elastic lamina, meaning that exercise shows the benefit roles in normalizing the increased vascular stiffness and decreased vascular distensibility in both small mesenteric arteries and coronary arteries [48]. However, high-intensity exercise leads to the opposite effects that increased oxidative stress, elevated blood pressure, and high vasoconstrictor activity are found [49]. Some studies report that males tend to achieve greater reductions than females from the exercise training [50]. However, the authors ignore the factors of menstrual cycle in the female subjects which affect the regulation of the autonomic nervous system [51]. Studying the effects of exercise on circadian rhythms using ambulatory blood pressure monitoring, there are significant reductions of daytime BP, but no obvious changes are observed at nighttime BP [44].

### 2.4. Blood Viscosity, Platelet Aggregation, and Thrombosis Profile

Blood viscosity in normal conditions is like a Newtonian fluid which is influenced by hematocrit, shear rate of blood flow, vascular caliber, and temperature. Elevated blood viscosity which is associated with blood resistance increases risks of cardiovascular complications. Blood viscosity is decreasing during exercise accompanied by decreased systemic vascular resistance [52]. More nitric oxide (NO) is produced attributed to greater shear stress in exercise and promote vasodilation [53, 54]. During exercise, erythrocyte volume is slightly increased; however, a much higher increase of plasma volume is generated which finally results in lower blood viscosity. Decline in plasma fibrinogen level is observed under the effect of exercise which plays important roles in declining erythrocyte aggregation and decreasing blood viscosity [55].

Platelet is a small volume component in blood which has no cell nucleus generated from megakaryocytes. Through forming thrombus, the platelets exert primary function of maintaining hemostasis of blood flow. Once an injury occurs, the platelets in the circulation will be activated and aggregated to the interrupted endothelial site to plug the hole [56]. So abnormality in platelet activation leads to a variety of atherosclerotic diseases mainly through excess thrombosis in small arteries like coronary arteries and blood vessels of the brain [57]. Exercise training presents an antithrombotic manner through platelet functional regulation. Through enhancing blood flow, exercise enhances endothelial NO production which counteracts platelet activation [58]. The moderate physical activity decreases both platelet adhesion and aggregation through downregulating intracellular calcium levels and increasing cGMP levels [59]. It is recommended by government guidelines that physical activity is an effective way to prevent thrombosis [60]. Exercise is also used to improve chronic complications of deep venous thrombosis, postthrombotic syndrome. A six-month exercise training markedly increases leg strength, hamstring and gastrocnemius flexibility, and overall fitness [61]. However, studies showed that the strenuous short-term exercise activated platelets and promoted aggregation of platelets, thus increasing the risk of MI or cardiac arrest. This suggests that acute exhausting exercise may trigger clot formation, but the mechanisms remain to be clarified [62].

### 2.5. Endothelial NO Production

NO is a gaseous signaling molecule playing an irreplaceable role in a variety of biological processes. It is catalyzed by various nitric oxide synthase (NOS) enzymes by using substrate L-arginine. Known as an endothelial-derived relaxing factor, NO contributes to not only endothelial-dependent relaxation (EDR) but also to the maintenance of endothelial function [63]. The endothelium is a single layer of cells in the intima of vessels separating blood from the tissue. The functions of the endothelium involve regulating angiogenesis, balancing vasoconstriction and vasodilation, adjusting smooth muscle cell proliferation, and excreting endocrine. The intact endothelium acts an indispensable role in vessel homeostasis [64]. In a cross-sectional study with 184 healthy individuals, L-arginine was reduced and production of NO was increased after exercise training [46]. The mechanisms may be partial that exercise training leads to increased blood flow and shear stress, contributing to endothelial NOS (eNOS) expression, NO release, and artery relaxation. NO activates soluble guanylate cyclase which increases cGMP and therefore activates protein kinase G (PKG). In blood vessels, PKG activation always induces relaxation and regulates blood pressure. In the heart, PKG works as a brake on stress response signaling [65]. Through increasing vascular AMPK/PPAR*δ*, exercise suppresses endoplasmic reticulum stress, thus increasing endothelial NO bioavailability. Exercise shows preserved EDR in the aorta and mesenteric artery in high-fat diet rats and db/db mice [66]. In the aging-induced downregulation of VEGF signaling cascade in the heart, exercise upregulates VEGF, its receptors Flt-1 and Flk-1, and the downstream signaling pathway Akt/eNOS [67]. Increased NO production usually facilitates angiogenesis and vascular permeability.

Dysfunction of EDR and decrease of cGMP in both plasma and vascular tissue are found in diabetic rats. However, chronic exercise improves endothelial function through releasing NO in the aorta. Interestingly, exercise does not affect the vascular function in normal rats administrated with L-arginine but improves vascular function in the aortas from diabetic rats [68]. In the high fat-induced obese mice, impaired EDR, reduced NO bioavailability, and decreased phosphorylation of eNOS are found in the coronary arteries which can be normalized by exercise. Through injecting *β*-adrenoceptor agonists, isoproterenol for 8 days, Yang recapitulated cardiac hypertrophy which was regarded as the primary risk factor for heart failure. After 4 weeks of exercise training, the eNOS expression did not change, but the phosphorylation of serine residue 1177 with an activating impact on eNOS was increased and the phosphorylation at threonine residue 495 with an inhibitory impact on eNOS was decreased. As a result, exercise promoted NO production and attenuated cardiac remodeling, echocardiographic and hemodynamic changes after *β*-adrenergic overload [69].

### 2.6. Leptin Sensitivity

Leptin is a 16 kDa circulating hormone which is predominantly released by adipocytes to exert regulation of food intake and energy metabolism. Through binding to leptin receptors on hypothalamic cells, leptin inhibits hunger, prevents weight gain, and promotes positive energy balance [70]. So leptin deficiency or leptin resistance promote diabetes, obesity, and other metabolic disorders. Exercise improves leptin resistance and sensitivity, attenuates body weight, and promotes homeostatic control of energy balance through influencing the leptin receptor in the ventromedial hypothalamic nucleus of obese mice [71, 72]. It also directly works on leptin receptors to induce NO-dependent vasodilation expressed in endothelial cells [73]. A decreased leptin sensitivity and hyperleptinaemia are found in obese mice coronary arteries, demonstrating leptin resistance and low leptin sensitivity. Exercise maintains leptin sensitivity of obese mice and preserves leptin receptor, thus exerting endothelial protection [74]. Exercise facilitates SOCS3 expression and improves leptin resistance in the liver and muscle of high-fat diet-induced rats [75]. Leptin sensitivity is restored by exercise manifested as facilitating fatty acid toward oxidation and away from triacylglycerol stores [76]. Through activating leptin, exercise also initiates the downstream JAK/STAT signal transduction pathway in obese mice to protect against myocardial ischemia-reperfusion injury [77]. Shapiro et al. found that leptin overexpression failed to reduce body weight in obese rats, and chronic leptin treatment aggravated the diet-induced obesity. Besides, wheel running for obese rats was insufficient to lower body weight. However, combinational administration of exercise and exogenous leptin dramatically induced weight loss and improved leptin sensitivity [78]. That means exercise may directly activate leptin signaling pathway or improve leptin sensitivity via coordinating with other therapeutic methods.

### 2.7. Modulation of Autonomic Function

The autonomic nervous system, regulated by the hypothalamus, consists of sympathetic nervous system, parasympathetic nervous system, and enteric nervous system. The primary autonomic functions include cardiac regulation, control of respiration, and vasomotor activity, which act largely unconsciously [79]. Exercise training has beneficial roles in autonomic function, as indicated by improved heart rate recovery (HRR) and heart rate variability (HRV) in various populations, such as in sedentary individuals and chronic heart failure patients [80]. HRR refers to the declining rate of heart rate and is recognized as an indicator of cardiac prognosis [81]. HRV is defined as consecutive heart beat variations in heart rate of sinus rhythm. Reduced HRV represents attenuation of autonomic regulation of sinoatrial node [82]. Exercise works as an intervention for autonomic dysfunction in type 2 diabetes by preserving HRV, HRR, and baroreflex sensitivity (BRS). BRS is regulated by sympathetic and parasympathetic autonomic nerves and is downregulated when there is cardiac autonomic neuropathy [83].

Electrocardiogram is a noninvasive way to determine cardiac conditions. Elevated R wave amplitudes and widened QT intervals are reliable predictors of autonomic neuropathy. Exercise training lowers heart rate and reduces QT interval and R wave amplitudes on electrocardiogram in the diabetic fatty rat model [84]. In myocardial-infarcted rats, structural remodeling leads to heterogeneity which causes slow conduction and creates the generation of arrhythmias. However, exercise training increases ratio of parasympathetic over sympathetic tones and decreases probability of ventricular arrhythmias of the MI rats [85]. The mechanism of exercise-induced improvement of arrhythmia might be related to intrinsic electrophysiological cardioadaptive mechanisms because of decreased action potential duration gradient between epicardial and endocardial cells in the exercise-trained rats [86]. The mechanisms by which exercise improves autonomic function and preserves neurovascular perfusion might be related to increasing NO bioavailability, lowering angiotensin II (AngII) levels, and suppressing chronic inflammation [87–90]. It is controversial that some studies show that MI, stroke, ventricular tachycardia, or fibrillation can be elevated during the progress of physical activity, so further studies about the duration, frequency, and intensity need to be specifically investigated.

### 2.8. Antioxidant Defense

ROS of physiological levels are responsible for signaling molecules to regulate normal physical activities. On the contrary, excessively generated ROS plays a crucial role in the initiation and progression of CVD [91]. ROS overproduction, decreased antioxidant enzymes, and the downstream targets damage the subcellular organelles, thus impairing the cardiovascular system. Excessive ROS may decrease NO bioavailability and NO/cGMP-dependent pathway which result in pathological vasoconstriction and hypertension [92]. ROS also initiates inflammation with the activation of redox-sensitive transcription factors and promotes expression of inflammatory molecules [93]. DNA is oxidized by ROS to form 8-oxo-dG which can be detected in plasma and urine [94]. AngII-induced insulin resistance is relevant to ROS, which can impair insulin signaling by decreasing phosphorylation of IRS1 and Akt and translocation of GLUT4 to the plasma membrane [24].

Exercise attenuates oxidative damage in the blood vessel, heart, brain, liver, skeletal muscle, and other organs. Exercise reduces age-related mitochondrial oxidative damage and increases mitochondrial NADH-cytochrome C reductase activity in the heart of aged rats [95]. By improving the antioxidant enzymes and redox status in many organs and pathologies, the chronic treadmill exercise has an anti-inflammatory effect. Exercise alleviates oxidative stress through inactivating ERK/AMPK signaling pathway and decreases expression of inflammatory factors like IL-6, high-sensitivity CRP, TNF*α*, and leucocyte differentiation antigens in diabetes [96]. In renovascular hypertensive rats, ROS released by vascular endothelial cells impairs EDR by decreasing NO production and facilitating vasoconstriction. Evidences indicate that exercise reduces ROS, improves endothelial function, and increases levels of elastin, fibrillin, and NO. The elevated cardiac MDA levels and MPO activity and depleted GSH and catalase expression in hypertensive rats are totally reversed by exercise training [97]. BRS is improved by physical activity through reducing oxidative stress in the rostral ventrolateral medulla to inhibit the sympathetic nerve [98, 99]. In diabetic rats, the overproduction of ROS leads to the abnormal aortic function and metabolic disorder. Through exercise training, enzymes producing ROS such as p67phox protein decrease and the enzymes taking part in scavenging oxygen-free radicals, like superoxide dismutase (SOD) 1, SOD2, catalase, and glutathione peroxidase (GPX), are normalized, even increased [48, 100]. That means, all the proteins taking part in oxidative stress tend to recover to normal levels after exercise treatment [101].

Exercise upregulates the antioxidant defense mechanisms through redox-sensitive transcription factors, NF-*κ*B and activator protein-1, and peroxisome proliferator-activated receptor gamma coactivator 1-alpha (PGC-1*α*). Exercise normalizes the aging-induced decline in mitochondrial oxidative capacity through upregulating PGC-1*α*, redox-sensitive transcription factors PPAR*γ*, and the target antioxidant genes like GPX and SOD2 [46, 102]. Through elevating laminar shear stress, exercise upregulates SOD and downregulates NADPH oxidase and VCAM-1. Through activating AMPK, exercise training increases sirt3 and PPAR*γ* and the targets SOD1, SOD2, GPX1, and catalase, thus resulting in less ROS production and more ROS clearance in skeletal muscles [103]. Exercise increases thioredoxin reductase 1 and decreases thioredoxin-interacting protein (TXNIP) in blood mononuclear cells and skeletal muscles, which promote antioxidant ability and eliminate free radical [104, 105]. TXNIP is a regulator of cellular redox signaling by binding thioredoxin to inhibit the neutralization of ROS, indirectly enhancing the oxidative stress [106]. Nuclear factor erythroid 2-like factor 2 (Nrf2) is reported to be an important transcription factor that performs antioxidant defense mechanisms through Nrf2-ARE signaling to activate antioxidant gene expression [107]. Mounting evidences demonstrate that the Nrf2-ARE signaling is downregulated in the aging heart, ischemia/reperfusion injury, and MI. Researchers find that through chronic exercise treatment, Nrf2 is upregulated and consequently activates the key antioxidant enzyme expression such as hemoxygenase-1, NADPH quinone oxidase-1, GPX1, GPX2, glutathione reductase, *γ*-glutamyl cysteine ligase-catalytic, *γ*-glutamyl cysteine ligase-modulatory, glucose-6-phosphate dehydrogenase (G6PD), and catalase in the heart [108, 109]. Based on these risk factors and studies, all major cardiovascular societies recommend that a minimum of 5 days a week of exercise, with at least 30 minutes of moderate-intensity aerobic activity, is needed to prevent CVD [110].

## 3. The Recommendations for Physical Activity

Because of low-cost, low-risk, and nondrug intervention, the European Society of Cardiology recommended that exercise training should be provided by cardiac rehabilitation programs in patients with non-ST elevation acute coronary syndrome in 2015 [111]. Physical activity is a part of everybody's life. However, the exercise intensity differs between people depending on the physical condition of individuals [112]. Although lots of studies show a positive correlation between exercise and good health, a thorough physical evaluation is necessary before an intensive exercise training program. The intensity, mode, duration, and frequency of exercise can strongly affect outcome.

Aerobic exercise is defined as using aerobic metabolism to extract energy in muscles, mainly referring to low- to moderate-intensity physical activities. As discussed above, aerobic exercise has favorable effects on lipid metabolism, cardiac remodeling, post-MI heart failure, insulin resistance, and endothelial function. Anaerobic exercise is an activity that synthesize energy sources without using oxygen as energy sources but glycolysis and fermentation. Anaerobic exercise usually refers to high-intensity training, including sprinting and power lifting. In several studies, high-intensity exercise is recommended to lower TG and LDL. Similar to aerobic exercise, anaerobic exercise also shows positive influences on body mass index and blood pressure [113]. In some cases, high-intensity training shows more beneficial effects on the cardiovascular system and EDR compared to low-intensity training [114]. The advantages of high-intensity intermittent exercise refer to the fact that the shorter time as 3-4 sessions/week will produce significant changes [115]. However, there is a paradox of disadvantages about anaerobic exercise training that the elevated mortality and sudden death are brought by high-intensity activity. A well-recognized viewpoint is that acute strenuous exercise increases the risks for cardiovascular diseases, like MI, by upregulating blood pressure [57]. In conclusion, intensive exercise should be intermittent especially for a long-term program. Professional supervision and guidance are indispensable when you conduct high-intensity exercise.

The general recommended exercise intensity for humans from the American Heart Association to prevent CVD is 30 minutes, 5 times a week to reach at least 150 minutes per week of moderate exercise, or 25 minutes, 3 times a week to reach at least 75 minutes per week of vigorous activity. Individuals can choose one way of physical activity or combine moderate and vigorous activities. They will also be benefited even if they divide the entire time into several parts of 10 to 15 minutes per day. For those who want to lower the risk for heart attack and stroke, 40 minutes of moderate to vigorous intensity aerobic activity, 3 or 4 times a week, is recommended [116]. Moderate-intensity exercise is more widely performed among people who are interested in exercise and enjoy rest time. Various studies show that the duration of physical activity but not intensity is the primary factor leading to benefits for humans [117].

Physical activity is regarded as an efficient way to prevent and counteract age-related changes in muscle and organic function [118]. As we know, any activity is better than none and it is never too late to start for the elders [119]. The American College of Sports Medicine and the American Heart Association recommend detailed description for elder people that 30 minutes, 5 times a week, of moderate intensity, or 20 minutes, 3 times a week, of vigorous intensity activity is good for aging-related diseases. Strength training should be included to enhance muscle groups and prevent falls as 8-10 exercises with 10-15 repetitions, twice a week [120]. For the elders, joining exercise classes to improve balance and prevent falls are strongly suggested [121]. Exercise is generally safe for older people unless for those elders who already suffered from health problems doing resistance training [122].

Examples of aerobic exercise include cycling, dancing, hiking, treadmill, climbing stairs, swimming, walking, and any activities (the criterion is that you can talk without breathing too hard). Anaerobic exercise refers to sprinting and power lifting accompanied by accumulation of lactic acid causing muscular fatigue [123]. However, the definition of moderate and vigorous exercise varies between individuals because the indispensability to measure the level of intensity bases on your fitness and overall health.

## 4. Future Perspectives

Many people are robbed by the thinking all-or-nothing when doing exercise. However, the low-level intensity training program could benefit you as long as you begin to change. Even the easiest activity is better than nothing and whenever you start is not too late [124].

There are also some limits for the exercise training when applied to real patients' treatment. The existing studies do not provide an exact guidance on the diverse intensity, duration, and frequency of exercise that may be suitable for the different kinds of diseases. In the future, personalization of exercise will be an irresistible trend. For those young and in healthy condition, it may be optional to consult a doctor before they start an exercise program. However, personalized exercise enables you to perform more specifically based on your present fitness level, interest, age, and gender [125]. For those old who have been inactive for a long period, it is necessary to consult physicians who will measure some indexes, like cardiorespiratory endurance, muscular strength, muscular endurance, and flexibility, and make graded exercise tests for individuals [126]. Also, for someone who has a baseline disease, the extreme exercise training seems to trigger the progression of disease. So tailored exercise would bring benefits not only for healthy humans but also for the patients with chronic disease [127]. For slow disease-related declines of muscle strength, tailored exercise may focus on the training to improve muscle strength and reduce the risk of falls. For those who has type 2 diabetes, body weight control and enhancement of peripheral circulation are the first two goals to realize. For people with arthritis, tailored exercise helps reduce joint stiffness and enhances muscle strength [125, 128]. The systematical and overall exercise guidance from a professional instructor is totally necessary. Personalization of exercise training will be a huge demand and replace the random mode of exercise today.

Although we get to know that exercise protects against CVD through attenuating sympathetic activity, arterial pressure, and heart rate, increasing blood flow and endothelial NO production, causing vessel dilation, decreasing inflammatory cytokine and reactive oxygen species formation, the exact mechanisms leading to transcriptional factor changes or transcriptional modifications are not studied. So future studies may be applied to the mechanisms of protective effects of exercise on the heart and vessels.
